# Automated, fast, robust brain extraction on contrast-enhanced T1-weighted MRI in presence of brain tumors: an optimized model based on multi-center datasets

**DOI:** 10.1007/s00330-023-10078-4

**Published:** 2023-08-24

**Authors:** Yuen Teng, Chaoyue Chen, Xin Shu, Fumin Zhao, Lei Zhang, Jianguo Xu

**Affiliations:** 1 Department of Neurosurgery, West China Hospital, Sichuan University, Chengdu, China; 2 Department of Radiology, West China Hospital, Sichuan University, Chengdu, China; 3 West China Hospital, No. 37, GuoXue Alley, Chengdu, 610041 People’s Republic of China; 4 College of Computer Science, Sichuan University, Chengdu, People’s Republic of China; 5 Department of Radiology, West China Second University Hospital, Sichuan University, Chengdu, China

**Keywords:** Deep learning, Brain extraction, Brain mask, Magnetic resonance imaging, Brain tumor

## Abstract

**Objectives:**

Existing brain extraction models should be further optimized to provide more information for oncological analysis. We aimed to develop an nnU-Net–based deep learning model for automated brain extraction on contrast-enhanced T1-weighted (T1CE) images in presence of brain tumors.

**Methods:**

This is a multi-center, retrospective study involving 920 patients. A total of 720 cases with four types of intracranial tumors from private institutions were collected and set as the training group and the internal test group. Mann–Whitney *U* test (*U* test) was used to investigate if the model performance was associated with pathological types and tumor characteristics. Then, the generalization of model was independently tested on public datasets consisting of 100 glioma and 100 vestibular schwannoma cases.

**Results:**

In the internal test, the model achieved promising performance with median Dice similarity coefficient (DSC) of 0.989 (interquartile range (IQR), 0.988–0.991), and Hausdorff distance (HD) of 6.403 mm (IQR, 5.099–8.426 mm). *U* test suggested a slightly descending performance in meningioma and vestibular schwannoma group. The results of *U* test also suggested that there was a significant difference in peritumoral edema group, with median DSC of 0.990 (IQR, 0.989–0.991, *p* = 0.002), and median HD of 5.916 mm (IQR, 5.000–8.000 mm, *p* = 0.049). In the external test, our model also showed to be robust performance, with median DSC of 0.991 (IQR, 0.983–0.998) and HD of 8.972 mm (IQR, 6.164–13.710 mm).

**Conclusions:**

For automated processing of MRI neuroimaging data presence of brain tumors, the proposed model can perform brain extraction including important superficial structures for oncological analysis.

**Clinical relevance statement:**

The proposed model serves as a radiological tool for image preprocessing in tumor cases, focusing on superficial brain structures, which could streamline the workflow and enhance the efficiency of subsequent radiological assessments.

**Key Points:**

*• The nnU-Net–based model is capable of segmenting significant superficial structures in brain extraction.*

*• The proposed model showed feasible performance, regardless of pathological types or tumor characteristics.*

*• The model showed generalization in the public datasets.*

**Supplementary information:**

The online version contains supplementary material available at 10.1007/s00330-023-10078-4.

## Introduction

Brain extraction, or skull stripping, refers to the process of removing skull and non-brain tissue in medical images. It is considered as a preliminary but important pre-processing step as its accuracy has a direct influence on the quality of subsequent image processing and the reliability of statistical analysis [1–6]. Manual segmentation of the brain is laborious, tedious, and time-consuming, commonly leading to significant inter- and intra-reader variations that may lead to analysis deviation [7]. With the development of deep learning methods in recent years, particularly in convolutional neural network (CNN) algorithms, automated methods have obtained state-of-the-art results in medical image segmentation [8–11]. However, the brain extraction models for cases present with brain tumors on contrast-enhanced T1-weighted (T1CE) magnetic resonance images (MRIs) should be further optimized to meet the demands from both clinicians and neuroimaging researchers.

Nowadays, the criteria defined by Eskildsen et al were widely used in previous studies. It is cited as follows: (a) inclusion of cerebrum, cerebellum, brainstem, and internal vessels and arteries, along with cerebrospinal fluid in ventricles, internal cisterns, and deep sulci; (b) exclusion of the skin, skull, eyes, dura mater, external blood vessels, and nerves [12], whereas, as the most important radiological examination for brain tumor evaluation, more information should be included in region of interest (ROIs) to provide a clear depiction of lesion location, boundary, and the relationship between tumor and adjacent structures.

Generally, the first concern is that the superficial structures on brain surface are not included in the mask (mainly referring to venous sinus and superficial vessels), while exclusion of these structures can significantly affect subsequent therapeutic decisions or oncological analysis [6, 13–16]. For example, in preoperative simulation that uses extracted brain for 3D reconstruction, tumoral invasion of structures on the brain surface is one of the major concerns for neurosurgeons that can significantly determine the surgical strategy [15, 17]. Exclusion of these structures may be inadequate and not appropriate. The second one is that all previous CNN models are trained and validated on datasets with only glioma cases [18–21]. As acknowledged by previous studies, the performance of supervised CNN models that were exclusively trained on scans of glioma subjects may be limited in cases with different types of brain tumors [18, 19]. Laborious manual correction and delineation may be still needed in these studies. The third one is that whether or to what extent can tumor characteristics influence model performance has not been answered yet. Obtaining the overall highest performance has become the primary objective in all previous studies, undermining other clinical concerns such as reliability, generalization, and convenience of a new method on tumor entities [18–22]. Previous methodological studies showed advanced performance with Dice similarity coefficients (DSCs) of more than 0.950, while the features of different types of brain tumors showed interspecific and intraspecific differences that may also lead to performance deviation [23, 24].

Therefore, to achieve the objective of clinical translation and widespread usage, a CNN model was developed with the latest state-of-art CNN architecture to perform automated brain extraction on T1CE MRIs in presence of brain tumors. Our model was optimized by including more brain surface structures in training, and by involving a multi-center dataset covering diversified tumor entities. Moreover, a series of intra-group analyses were performed to investigate if our model could feasibly segment brain images regardless of tumor characteristics.

## Materials and methods

### Datasets

**Fig. 1:**
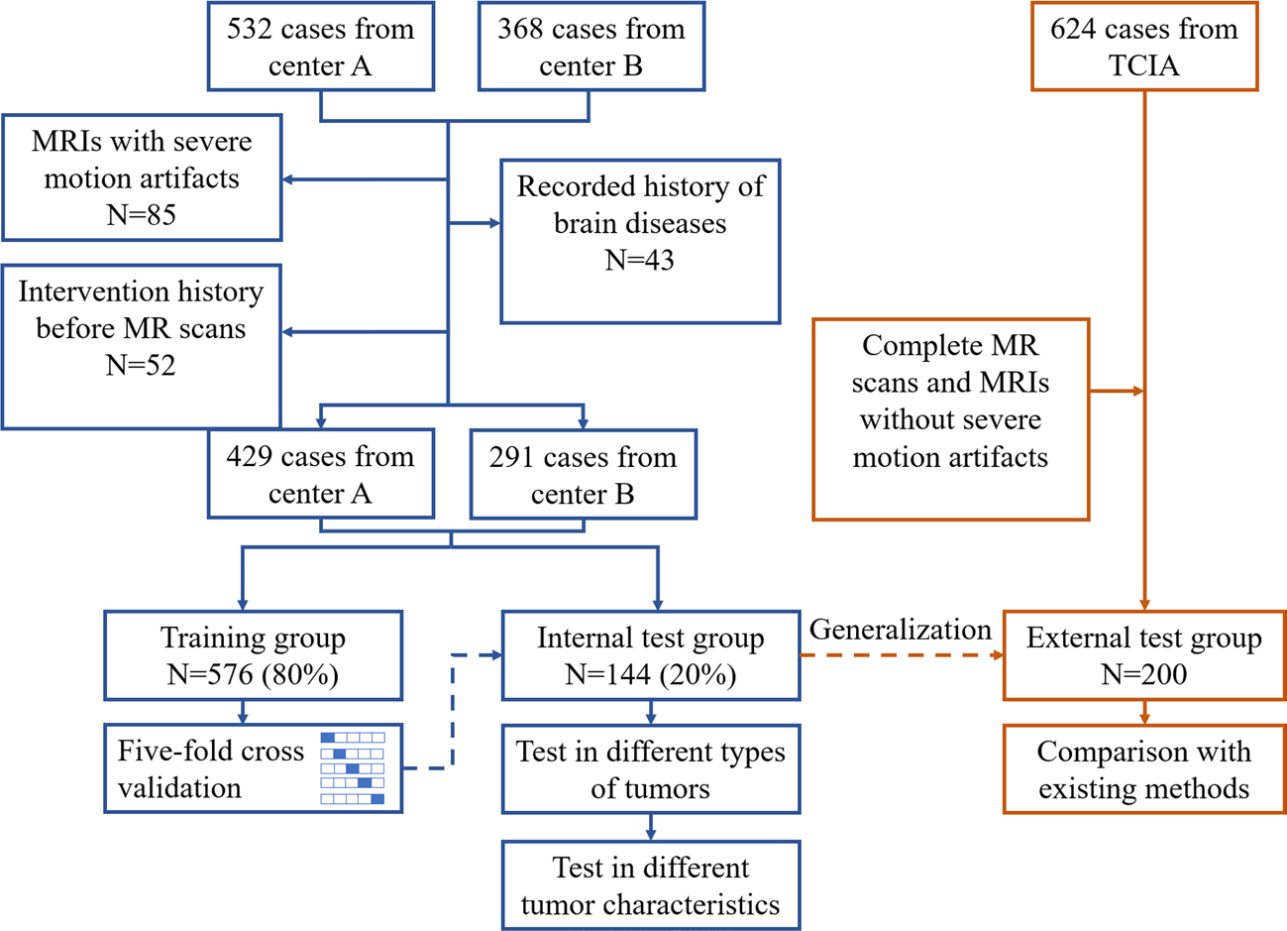
Flowchart shows the participants selection from internal and external groups. TCIA, The Cancer Imaging Archive. MRIs, magnetic resonance imagings

This was a retrospective, multi-center research. Figure 1 shows the flowchart of patient selection. In private center A and center B, 532 cases and 368 cases were initially selected from the radiological department between January 2016 and December 2021. All of the cases underwent standard pre-treatment magnetic resonance scans and received surgical resection in our institutions. In total, 180 patients were excluded based on the following exclusion criteria: (1) MRIs with severe motion artifacts (*N* = 85); 92) intervention history before MR scans, such as biopsy and radiotherapy (*N* = 52); 93) recorded history of other brain diseases, such as hypertensive intracerebral hemorrhage (*N* = 43). Eventually, 429 cases from center A and 291 cases from center B were included in the current study.

The T1CE images closest to clinical intervention were collected from the Picture Archiving and Communication System (PACS), including three orientations of axial, sagittal, and coronal views. Clinical features and radiological features were also collected and interpreted, including age, gender, pathological diagnosis, and imaging findings. Detailed MR scanning parameters are shown in Supplemental Material 1.

As for public datasets, a total number of 200 patients with complete, high-quality MR scans were randomly selected from three The Cancer Imaging Archive (TCIA) datasets, including The Cancer Genome Atlas Glioblastoma (TCGA-GBM) [25], The Cancer Genome Atlas Lower Grade Glioma (TCGA-LGG) [26], American College of Radiology Imaging Network (ACRIN) 6684 [27], and Vestibular-Schwannoma-SEG [28].

### Definition of brain mask and ground truth manual segmentation

We defined the following criteria for ground truth mask: (a) including all cerebral and cerebellar gray and white matter, brainstem, cerebrospinal fluid in the ventricles, and the cerebellar cistern, lesion sites in the brain, superficial venous sinuses (sagittal sinus and transverse sinus), and (b) excluding the skin, skull, eyes, dura mater, cavernous sinus area, and exterior blood vessels and nerves (such as carotid arteries and optic chiasm).

Manual segmentation was performed using 3D Slicer software [29]. The brain masks were segmented by five experienced neuroradiologists with more than 10 years of experience in image reading. Following the instructions of software, the mask was delineated on three orientations in consensus reading, followed by reviewing and correction by two senior neuroradiologists (F.M.Z. and J.G.X., with more than 20 years of experience in image reading). To examine intra-observer repeatability, thirty cases were randomly selected and segmented again with at least 15-day interval.

### Deep learning model for automated brain extraction

The latest state-of-art segmentation CNN network, nnU-Net, was used for modeling. It is an advanced CNN network containing a deep learning–based self-configuration module that can automatically configure image pre-processing, network architecting, cross-validation training, and post-processing [30]. The 3D full-resolution U-Net model was determined as the optimal model architecting, and the configuration of the model is provided in Supplemental Material 2. As shown in Fig. 1, the cases from private datasets were randomly divided into the training group and the internal test group in a ratio of 4:1. A fivefold cross-validation strategy was used in model training.

Public datasets were used as the independent test group, where the generalization of trained model was tested. We also validated the performance of existing methods on these TCIA datasets, including Robust Learning-Based Brain Extraction (ROBEX), HD-BET, and Brain Mask Generator (BrainMaGe) [18, 19, 31]. All experiments were written in Python language, and were performed in the machine equipped with four NVIDIA 3090 data center accelerator.

### Evaluation metrics and statistical analysis

The imaging findings were interpreted by two senior radiologists (F.M.Z. and J.G.X.). The CNN models’ performance was evaluated by comparing the ground truth and the prediction. A series of commonly used metrics were calculated, including median DSC, false-negative rate (FNR), and false-positive rate (FPR). Two metrics were also introduced to make a better demonstration of model performance in segmenting brain surface, including Hausdorff distance (HD), for measuring the maximal contour distance (mm) between the ground truth masks and the predictions, and mean surface distance (MSD), for measuring the average distance (mm) between two masks’ boundaries.

Categorical variables were presented with frequencies and percentages, and continuous variables were presented with medians and interquartile range (IQR). Kolmogorov–Smirnov test, Mann–Whitney *U* test (*U* test), and Wilcoxon signed rank test (Wilcoxon test) were carried out in intra-group analysis, as appropriate. Statistical analysis was performed with GraphPad Prism. *p* < 0.05 implicated statistical significance. 3DMeshMetric was used to visualize the spatial distribution of errors between the ground truth mask and the prediction. Volume rendering of 3D brain image was performed by using composite with shading technology without surface smoothing.

## Results

### Patient characteristics

A total number of 720 patients (720 exams) were collected from private institution A and institution B. The mean age of patients was 53 years old, and the sex ratio was male:female = 163:197. As for the pathological distribution, 505 cases were diagnosed with meningioma, 50 cases with low-grade glioma, 78 cases with high-grade glioma, and 87 cases with vestibular schwannoma. For the 200 cases collected from the public dataset, twenty-one cases were present with low-grade glioma, 79 cases were with high-grade glioma, and 100 cases were with vestibular schwannoma. The clinical characteristics of the internal and external datasets are represented in Table 1.

**Table 1:** Clinical, histopathological, and radiological characteristics of cases from internal and external datasets

	Internal dataset	External dataset	*p* value
No. of patients	720	200	-
Pathological type	Meningioma (*N* = 505)	Low-grade glioma (*N* = 21)	< 0.001
	Low-grade glioma (*N* = 50)	High-grade glioma (*N* = 79)	
	High-grade glioma (*N* = 78)	Vestibular schwannoma (*N* = 100)	
	Vestibular schwannoma (*N* = 87)		
Gender			
Male	326 (45.3%)	Not provided	-
Female	394 (54.7%)		
Mean age (range)	53 ± 13 (9–81)	Not provided	-
Skull base tumor	371 (51.5%)	110 (55.0%)	0.384
Peritumoral edema (PE)	255 (35.4%)	77 (38.5%)	0.422
Invaded venous sinus (IVS)	240 (33.3%)	14 (7.0%)	< 0.001

Data in parentheses are percentages

### Model performance in internal test

**Fig. 2:**
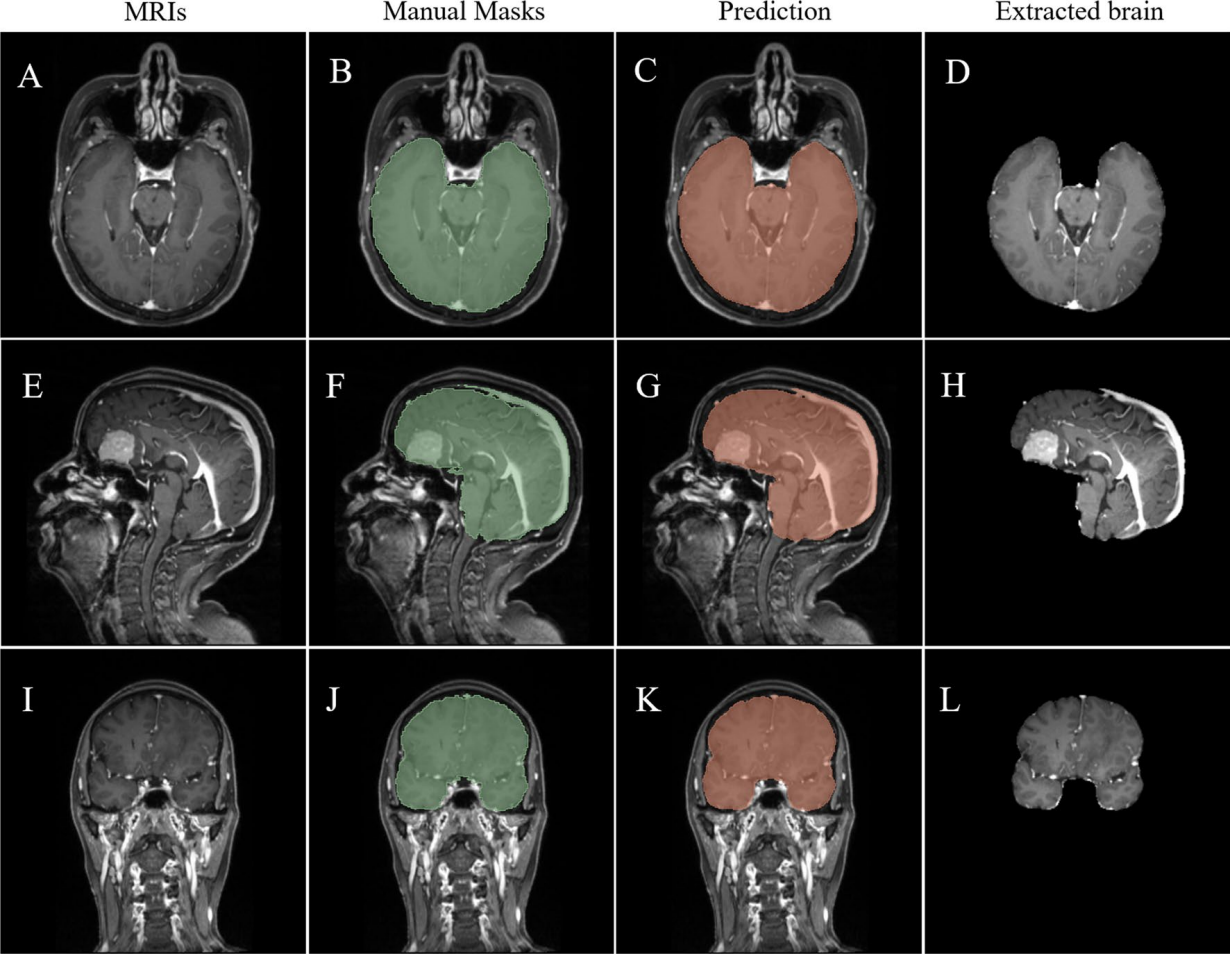
A brain extraction example for skull base meningioma from internal test. **A**–**D** Axial views. **E**–**H** Sagittal views. **I**–**L** Coronal views. Segmentation performance of this case is DSC of 0.991, HD of 5.196 mm. MRIs, magnetic resonance imagings

Overall, the automated model performed well in the internal test, with median DSC of 0.989 (IQR, 0.988–0.991), FNR of 0.012 (IQR, 0.009–0.015), FPR of 0.008 (IQR, 0.006–0.012), HD of 6.403 mm (IQR, 5.099–8.426 mm), and MSD of 0.013 mm (IQR, 0.011–0.015 mm). Model segmentation performance of cases from the internal test group is shown in Fig. 2 and Supplemental Material 3a.

The models’ performance regarding pathological type is presented in Table 2. In particular, the model exhibited slightly inferior but statistically significant performance in the meningioma dataset, with a median DSC of 0.989 (IQR, 0.987–0.990, *p* < 0.001) and a median HD of 7.000 mm (IQR, 5.477–9.165 mm, *p* = 0.001). Similar outcomes were observed in the vestibular schwannoma cohort, which represents another type of extra-axial tumor, with a median DSC of 0.990 (IQR, 0.990–0.991, *p* = 0.027) and a median HD of 5.385 mm (IQR, 4.243–6.708 mm, *p* = 0.010). However, there was no significant difference in the low-grade and high-grade glioma groups. The box chart and heat map of this intra-group analysis are shown in Supplemental Material 4a and 4b.

**Table 2:** Performance of the model in different pathological types of tumor groups

	DSC	*p* value	HD (mm)	*p* value
Meningioma	0.989 (IQR, 0.987–0.990)	< 0.001	7.000 (IQR, 5.477–9.165)	0.001
Low-grade glioma	0.991 (IQR, 0.990–0.991)	0.094	5.916 (IQR, 4.354–8.475)	0.294
High-grade glioma	0.991 (IQR, 0.987–0.991)	0.132	5.521 (IQR, 4.687–7.849)	0.124
Vestibular schwannoma	0.990 (IQR, 0.990–0.991)	0.027	5.385 (IQR, 4.243–6.708)	0.010

The *p* values indicate if there is the statistical significance of the model performance when comparing one type of tumor to others. The *p* value is computed by comparing the DSC and HD for one type of tumor with all other three types of tumors put together

*DSC* Dice similarity coefficient, *HD* Hausdorff distance, *IQR* interquartile range

### Model performance regarding radiological characteristics

Among the cases evaluated, 35.4% presented with peritumoral edema (PE). As demonstrated in Table 3, results from the *U* test revealed a significant disparity in the PE group, with a median DSC of 0.990 (IQR, 0.989–0.991, *p* = 0.002), and a median HD of 5.916 mm (IQR, 5.000–8.000 mm, *p* = 0.049). Invaded venous sinus (IVS) was detected in 240 cases (33.3%), and 51.5% of tumors were located in the skull base. However, intra-group analysis indicated non-significant results in cases with IVS (DSC = 0.990 (IQR, 0.988–0.991, *p* = 0.124), HD = 6.000 mm (IQR, 5.000–8.062 mm, *p* = 0.155)), or in cases with skull base tumors (DSC = 0.989 (IQR, 0.987–0.990, *p* = 0.553); HD = 7.211 mm (IQR, 5.385–8.775 mm, *p* = 0.398)). Supplemental Material 4c shows the box plot that indicates the intra-group analysis to investigate whether model performance is related to tumor characteristics.

**Table 3:** nnU-Net model performance regarding tumor characteristics

	Internal test			
	DSC	*p* value	HD (mm)	*p* value
Skull base tumor	0.989 (IQR, 0.987–0.991)	0.553	6.083 (IQR, 5.196–8.306)	0.398
Non-skull base tumor	0.990 (IQR, 0.988–0.991)		7.000 (IQR, 5.099–8.702)	
Peritumoral edema (PE)	0.990 (IQR, 0.989–0.991)	0.002	5.916 (IQR, 5.000–8.000)	0.049
Non-peritumoral edema	0.989 (IQR, 0.987–0.991)		6.708 (IQR, 5.385–8.860)	
Invaded venous sinus (IVS)	0.990 (IQR, 0.988–0.991)	0.124	6.000 (IQR, 5.000–8.062)	0.155
Non-invaded venous sinus	0.989 (IQR, 0.988–0.991)		6.557 (IQR, 5.196–8.630)	

*DSC* Dice similarity coefficient, *HD* Hausdorff distance, *IQR* interquartile range

### Model generalization

**Fig. 3:**
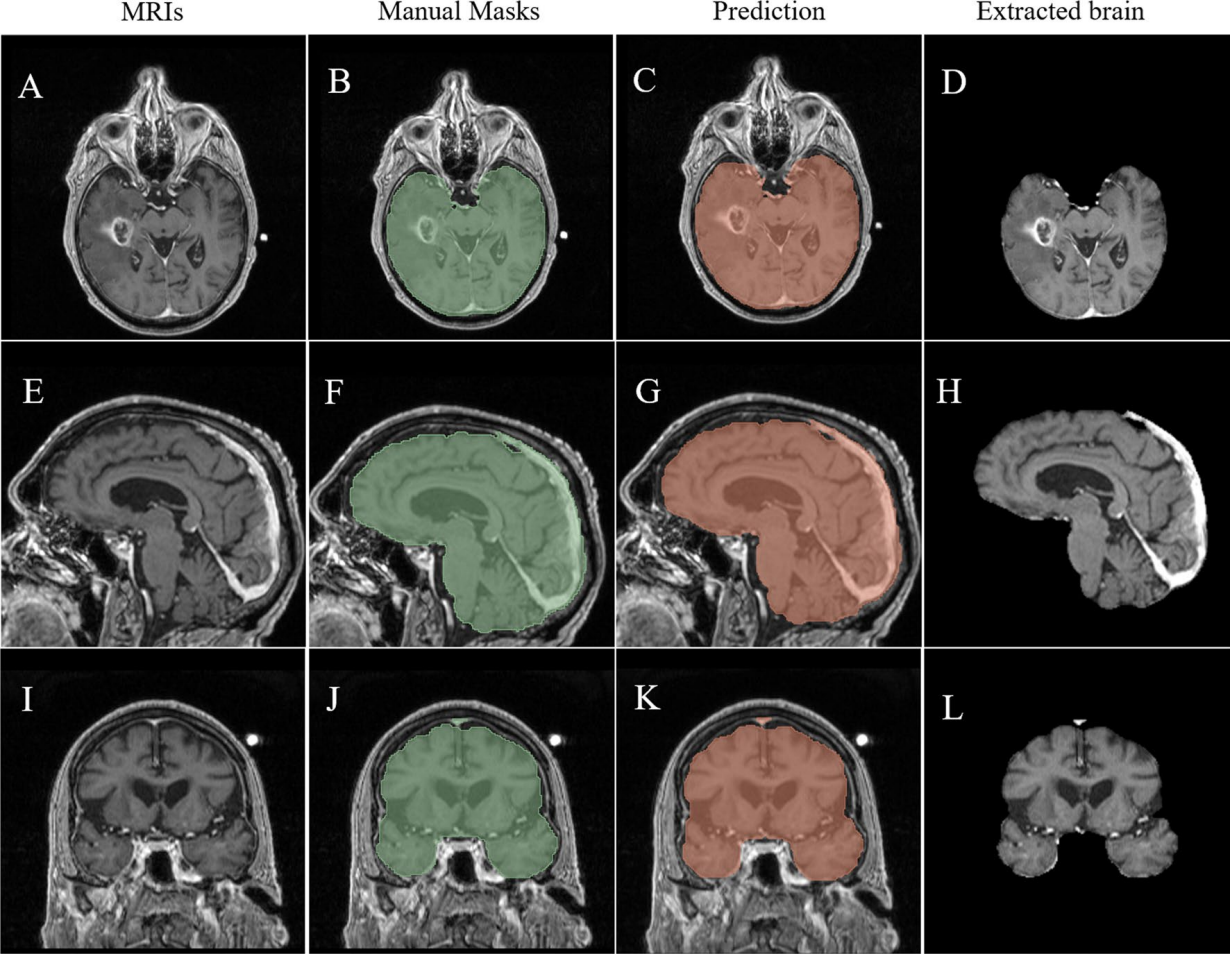
A brain extraction example for temporal glioblastoma from external test. **A**–**D** Axial views. **E**–**H** Sagittal views. **I**–**L** Coronal views. Segmentation performance of this case is DSC of 0.984, HD of 7.681 mm. MRIs, magnetic resonance imagings

The model achieved robust generalization in the independent datasets with median DSC of 0.991 (IQR, 0.983–0.998), FNR of 0.003 (IQR, 0.000–0.011), FPR of 0.008 (IQR, 0.004–0.020), HD of 8.972 mm (IQR, 6.164–13.710 mm), and MSD of 0.013 mm (IQR, 0.006–0.022 mm). Predictions of good and poor examples from the external test group are shown in Fig. 3 and Supplemental Material 3. However, the model exhibited significantly lower performance in the glioma dataset compared to the vestibular schwannoma group in terms of Dice similarity coefficient (DSC) (0.983 vs 0.998, *p* < 0.001) and Hausdorff distance (HD) (7.000 mm vs 12.860 mm,* p* < 0.001).

### Performance of the existing models

In the internal test, the DSCs of HD-BET, BrainMaGe, Robex were 0.989 (IQR, 0.980–0.993), 0.961 (IQR, 0.945–0.970), and 0.957 (IQR, 0.948–0.963), respectively. The HDs were 9.165 mm (IQR, 7.681–11.550 mm), 27.330 mm (IQR, 12.240–53.920 mm, *p* < 0.001), and 12.530 mm (IQR, 11.000–14.20 mm, *p* < 0.001), respectively. Similar results were also suggested in the external test. The existing models’ performance is summarized in Table 4, and more detailed evaluation metrics regarding tumor pathological types are shown in Supplemental Material 5.

**Table 4:** Three existing brain extraction models’ performance in internal and external tests

	Model	DSC	HD
Internal test	HD-BET	0.989 (IQR, 0.980–0.993, *p* = 0.111)	9.165 mm (IQR, 7.681–11.550 mm, *p* < 0.001)
	BrainMaGe	0.961 (IQR, 0.945–0.970, *p* < 0.001)	27.330 mm (IQR, 12.240–53.920 mm, *p* < 0.001)
	Robex	0.957 (IQR, 0.948–0.963, *p* < 0.001)	12.530 mm (IQR, 11.000–14.20 mm, *p* < 0.001)
External test	HD-BET	0.983 (IQR, 0.978–0.985,* p* < 0.001)	10.650 mm (IQR, 9.000–12.750 mm, *p* = 0.001)
	BrainMaGe	0.951 (IQR, 0.920–0.967, *p* < 0.001)	31.520 mm (IQR, 10.050–42.660 mm, *p* < 0.001)
	Robex	0.959 (IQR, 0.951–0.973, *p* < 0.001)	12.850 mm (IQR, 11.140–14.470 mm, *p* < 0.001)

The *p*-value is computed by comparing the DSC and HD for one type of model with all other three types of models put together

*DSC* Dice similarity coefficient, *HD* Hausdorff distance, *IQR* interquartile range

### Automated 3D brain surface rendering

**Fig. 4:**
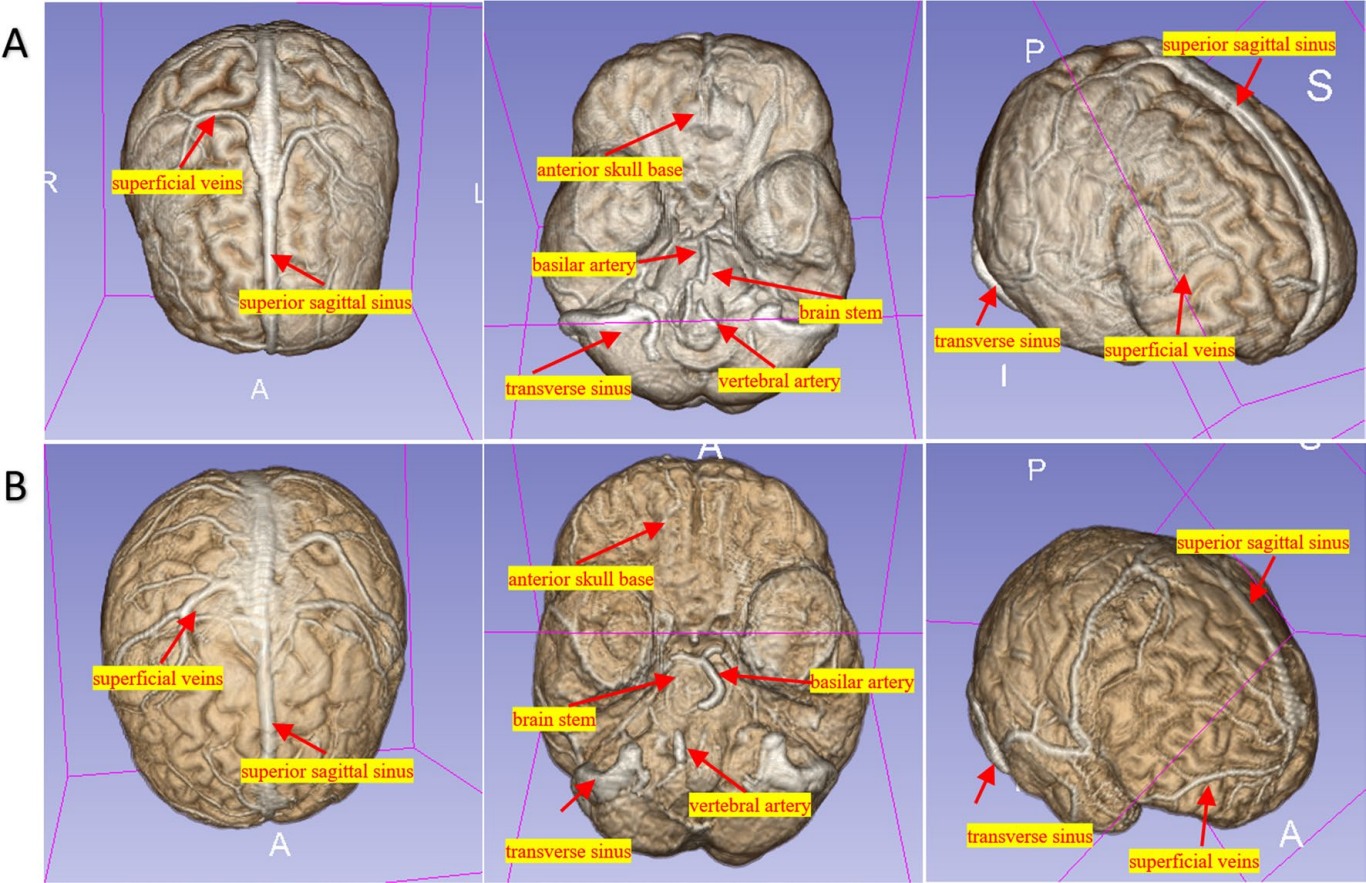
Two examples of 3D reconstructions based on brain masks by the 3D Slicer, with above, bottom, and lateral anterior views from internal (**A**) and external (**B**) tests

**Fig. 5:**
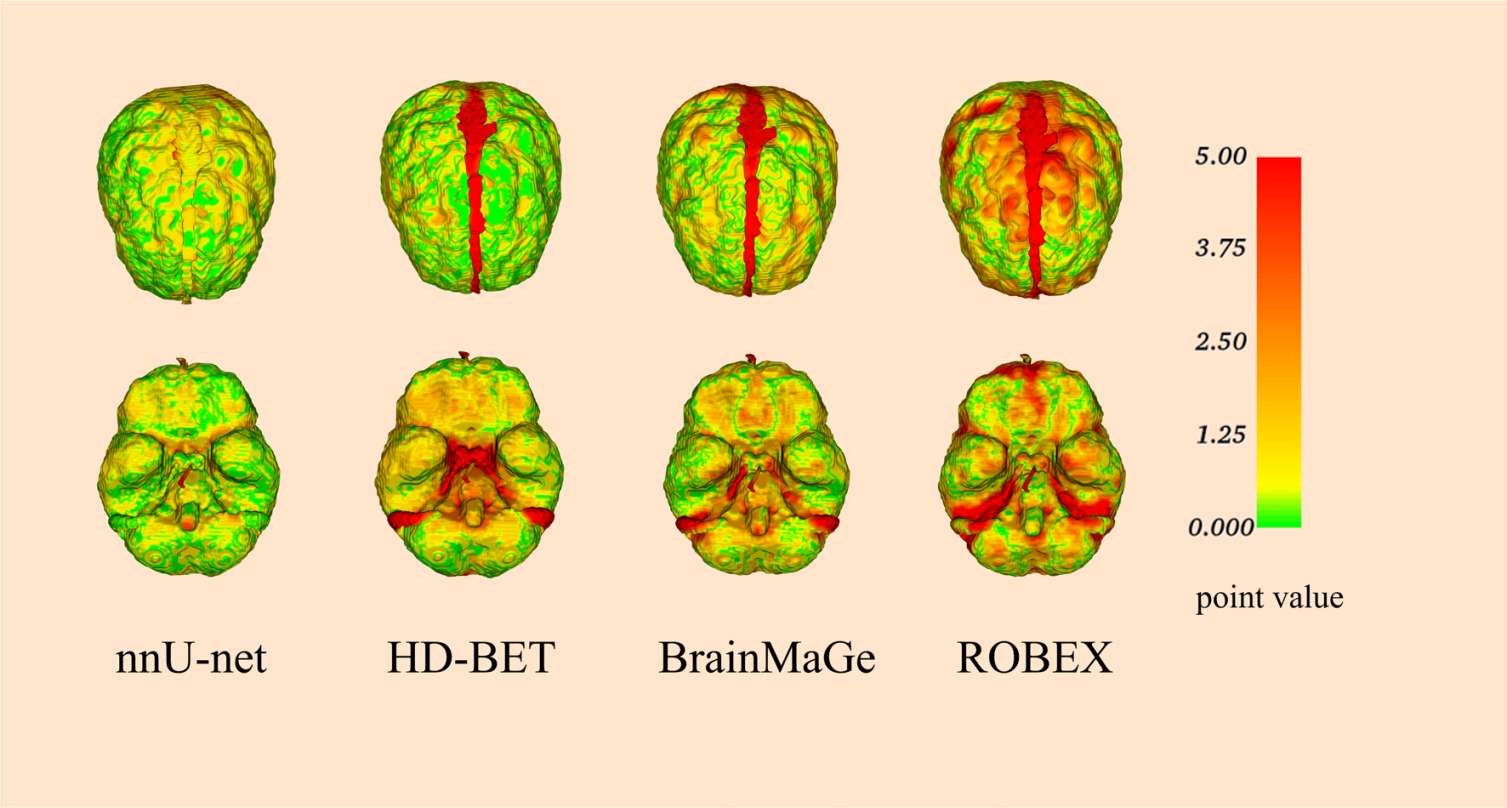
Error distribution map was formed by comparison between ground truth mask and prediction of each model. Qualitative and quantitative were assessed by point value, and the points range was set from 0 to 5.00. The color in the figure corresponds to the point error (the red area represents a large error, while the green area represents a small error)

Figure 4 shows the samples of 3D brain images using the mask generated by the nnU-Net model. The overall review of brain was shown. Important structures were well displayed, including superior sagittal sinus, transverse sinus, superficial vein, anterior skull base, brain stem, vertebral artery, and basilar artery. The nnU-Net model showed relatively low error rates in the segmenting skull base, superior sagittal sinus, transverse sinus, and brain stem (Fig. 5).

### Intra-observer reproducibility, and comparison of radiologists and models

For each brain mask, it took approximately 1 h and 20 min for the manual procedure of delineation, check, and correction, while the nnU-Net model took 4 min to perform inference on both CPU and GPU. As for the randomly selected 30 patients, Bland–Altman plots suggested that there was good intra-observer reproducibility of manual segmentation (Supplemental Material 6A). Bland–Altman plots also indicated that the automated method performed as well as expert manual segmentation despite lesion characteristics, as presented in Supplemental Material 6B and Supplemental Material 6C.

## Discussion

In the present study, an automated model utilizing deep learning technology was developed to achieve rapid and robust brain extraction on T1CE MRIs in the presence of intracranial tumors. Our model confers an advantage in that it incorporates superficial structures into the brain extraction mask, a crucial factor in oncological analysis that had been previously unaddressed in other studies. The proposed method is applicable to a wide range of MRI hardware and acquisition parameters commonly encountered in both clinical and research practice. The model was trained on a large multi-center MRI dataset and subsequently tested for generalizability on three independent public datasets, resulting in DSCs of over 0.980.

Compared to previous methodological studies (summarized in the Supplemental Material 7), the present study was more clinically relevant, addressed several shortcomings concerning clinicians, and improved the model performances with the latest CNN network. First, we improved the brain extraction criterion by incorporating superficial brain structures and fine-tuned model performance on these regions, which were used during model training. Unlike other imaging sequences, for neurosurgeons and neuro-radiologists, who might primarily rely on a CNN model for brain extraction on T1CE MRIs, discerning the anatomical correlation between tumors and adjacent structures is a crucial aspect of clinical decision-making [15]. Previous studies failed to address this issue, resulting in poor model performance in these areas. Secondly, we evaluated the model’s performance on four different types of tumors. Although previous studies have reported promising results, none has tested the trained model on a dataset that covers multiple types of contrast-enhanced images of tumors. In contrast, our model was trained and tested on both intra-axial and extra-axial tumors, and the good performance indicated its good generalizability. Thirdly, we conducted an intra-group analysis to examine whether the model could be generalized despite the diverse image patterns of brain tumors. Finally, we assessed the generalizability of our model on four external datasets that were independent and publicly available. All of these improvements have enhanced the practicality of our model and expanded the potential for its clinical translation and widespread usage.

Shape fidelity of the automated segmentation outline to the true brain mask is very important. While the DSC is commonly used as a metric for evaluating segmentation performance, it is insensitive to differences in edges that have a small volumetric effect relative to the total volume. Therefore, we combined the DSC with the HD in our intra-group analysis. In this analysis, we found some intriguing results that require further clarification. Overall, the model demonstrated promising performance with median DSCs exceeding 0.980 in each group, despite the distinct appearances of the four tumor entities. However, in the internal dataset, we observed slightly decreasing DSCs in the meningioma and vestibular schwannoma groups, whereas in the external dataset, the model exhibited an increase in HD in the vestibular schwannoma group. These two extra-axial tumors originate in the meninges and cranial nerves, leading to severe structural abnormalities that may account for the decreased model performance. Additionally, the results suggested that the model performed better in tumors presenting with PE, potentially because the swollen cortex displayed a darkening intensity, increasing the contrast between the brain and enhanced meninges and thereby facilitating segmentation. Although the statistical performance remained feasible, these findings are significant, as manual correction may be necessary in such cases.

Our research has several limitations. First, it was a multicenter, retrospective study with inherent selection bias. Second, compared with previous studies, only contrast-enhanced images were used, and other sequences, including T1WI, T2WI, and FLAIR, were not involved in our research. Image co-registration may be required if researchers want to perform skull stripping in these sequences. Third, our study lacked methodological novelty in terms of the used CNN structure. Although the network architecture used in this study was based on the classic nnU-net framework, its performance was remarkable, and we did not feel it necessary to optimize the network further. Fourth, due to ethical constraints, the model was solely trained and evaluated on cases with tumors. Therefore, additional studies are required to confirm the generalizability of our results.

In conclusion, we have presented a novel fully automatic deep learning model for brain extraction on T1CE MR scans. The proposed model enables extraction of the brain with tumor and provides more detailed information about the brain surface. Our study demonstrates that the model has a high level of performance and generalization in the segmentation task, which could potentially alleviate the workload of radiologists and offer a valuable tool for future neuroimaging research and oncological studies.

### Supplementary Information

Below is the link to the electronic supplementary material.

Supplementary file1 (DOCX 2537 KB)

## Data Availability

The MR images or related data were not allowed to be shared publicly as privacy-protection policy, whereas we would like to share the brain-extraction model in the Git-hub (https://github.com/XU-NET/BEIT).

## Abbreviations

ACRIN: American College of Radiology Imaging Network
BrainMaGe: Brain mask generator
CE: Cross-entropy
CNN: Convolutional neural network
DCS: Dice similarity coefficient
FNR: False-negative rate
FPR: False-positive rate
HD: Hausdorff distance
IQR: Interquartile range
IVS: Invaded venous sinus
MRI: Magnetic resonance image
MSD: Mean surface distance
PACS: Picture Archiving and Communication System
PE: Peritumoral edema
ROBEX: Robust Learning-Based Brain Extraction
ROIs: Region of interest
T1CE: Contrast-enhanced T1-weighted
TCGA-GBM: The Cancer Genome Atlas Glioblastoma
TCGA-LGG: The Cancer Genome Atlas Lower Grade Glioma
TCIA: The Cancer Imaging Archive
U test: Mann-Whitney *U* test
Wilcoxon test: Wilcoxon signed rank test

## Declarations

### Guarantor

The scientific guarantor of this publication is Jianguo Xu.

### Statistics and biometry

No complex statistical methods were necessary for this paper.

### Study subjects or cohorts overlap

No.

### Methodology

• retrospective

• experimental

• multicenter study
