# Supplementary material for: Automated, fast, robust brain extraction on contrast-enhanced T1-weighted MRI in presence of brain tumors: an optimized model based on multi-center datasets
Source: Eur Radiol. 2023 Aug 24;34(2):1190–9. doi: 10.1007/s00330-023-10078-4 (PMC10853304; doi:10.1007/s00330-023-10078-4)
Supplement: Supplementary file 1 — Supplementary file1 (DOCX 2537 KB) [file 330_2023_10078_MOESM1_ESM.docx]

**Automated, fast, robust brain extraction on contrast-enhanced T1-weighted MRI in presence of brain tumors: an optimized model based on multi-center datasets**

**ELECTRONIC SUPPLEMENTARY MATERIAL**

**Supplemental Material 1** MR scanning parameters of cases from private institutions. MR scans in two institutions were performed in the head-first supine position. Gadopentetate dimeglumine (dose: 0.1 mmol/kg) was used as contrast agent.

|  | center A (3.0 T) | center B (1.5T) | center B (3.0 T) |
| --- | --- | --- | --- |
| Scanner | SIEMENS Trio Tim | SIEMENS Symphony P | SIEMENS Skyra |
| Slice Thickness (mm) | 1 | 1.1 | 1 |
| Repetition Time (ms) | 1550 | 2050 | 1550 |
| Echo Time (ms) | 1.98 | 2.65 | 2.44 |
| Echo Number (s) | 1 | 0 | 1 |
| Percent Phase Field of View | 90.625 | 68.75 | 75 |
| Acquisition Matrix | 0\256\232\0 | 0\256\158\0 | 0\256\154\0 |
| Flip Angle (°) | 9 | 12 | 8 |

Note.—Data in parentheses are magnetic field strengths.

**Supplemental Material 2** Implementation details of nnU-Net model.

In image pre-processing, after cropping the peripheral area with pixel values equaling to zero, all images and manual segmentation masks were adaptively resampled to a median image size of 254*254*210 voxels, followed by intensity normalization by z-scoring. Target spacing of the model was 1.000*0.957*0.898mm, patch size was set as 128*128*128 voxels, and batch size was set to 2. Data augmentation techniques were also applied, including rotations, gamma correction, mirroring, scaling, Gaussian noise, Gaussian blur, brightness, contrast and simulation of low resolution.

The networks were trained for 1,000 epochs, with one epoch being fed with 250 mini-batches. Stochastic gradient descent with Nesterov momentum was set to 0.99 as optimizer. Initial learning rate was set as 0.1, and would reduce in training by using the ‘poly’ learning rate policy for learning network weights. The loss function was the sum of cross-entropy (CE) and Dice loss to improve training stability and segmentation accuracy. The calculation formulas of the loss function are:

$$L_{total}=L_{Dice}+L_{CE}$$

$$L_{Dice}=-\frac{2\sum_{i} u_{i}v_{i}}{\sum_{i} u_{i}+\sum_{i} v_{i}}$$

$$L_{CE}=\sum v_{i}\log u_{i}+\left( 1-v_{i} \right)log\left( 1-u_{i} \right)$$

The v represents the ground truth，u represents the predicted value.

Connected component-based post-processing was determined as necessary to eliminate the false positives by removing all but the largest connected component. Six-connectivity is used to determine the largest connected component.

**Supplemental Material 3** The best and the worst segmentation examples in internal and external test. (A-C) The best case in the internal test, with DSC of 0.993 and HD of 3.741mm. (D-F) The best case in the external test, with DSC of 0.999 and HD of 4.000mm. (G-I) The worst case in the internal test, with DSC of 0.975 and HD of 31.161mm. False positive in the posterior cranial fossa region. (J-L) The worst case in the external test, with DSC of 0.915 and HD of 19.748mm. False positive in the parietal region.


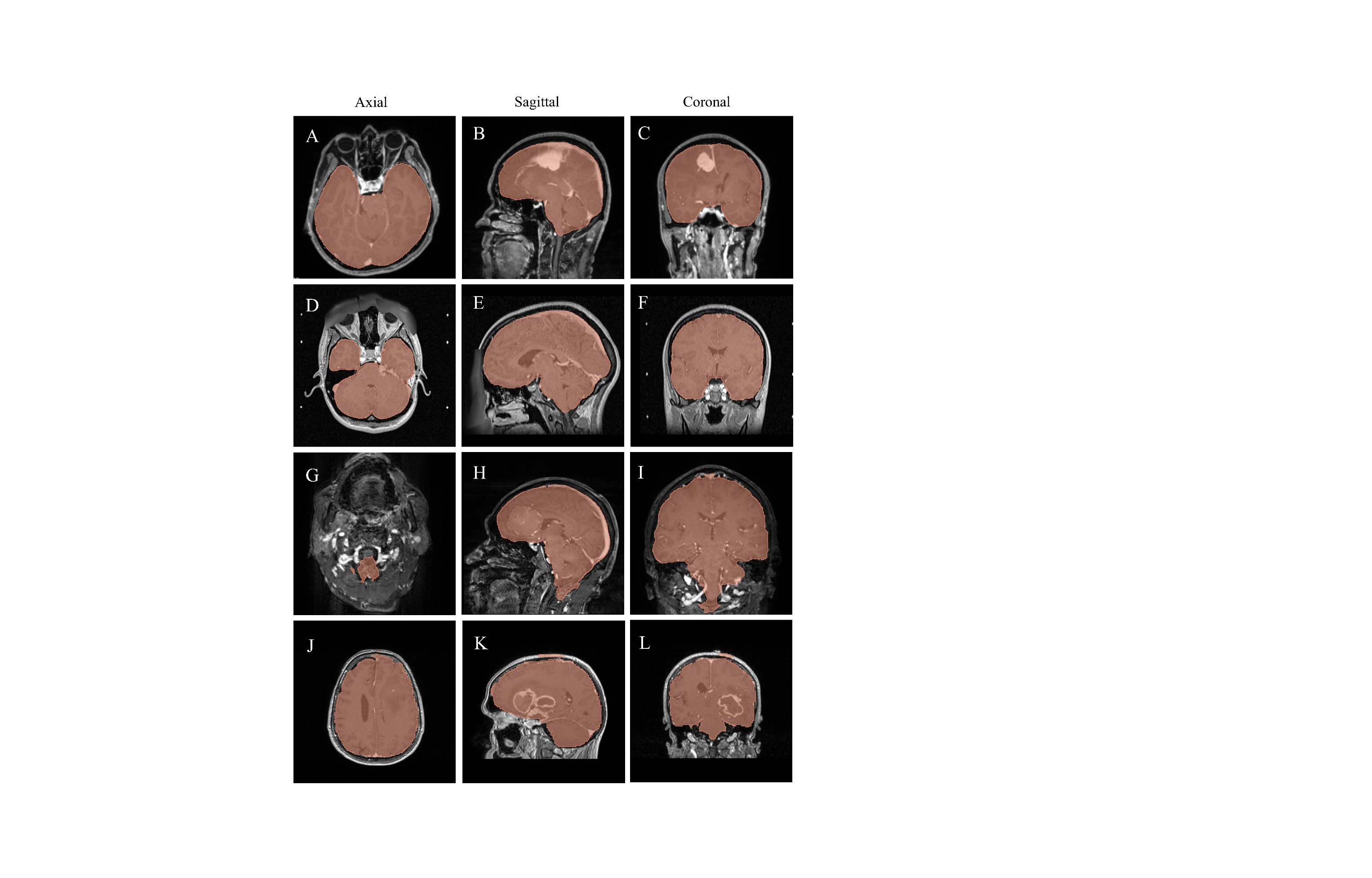


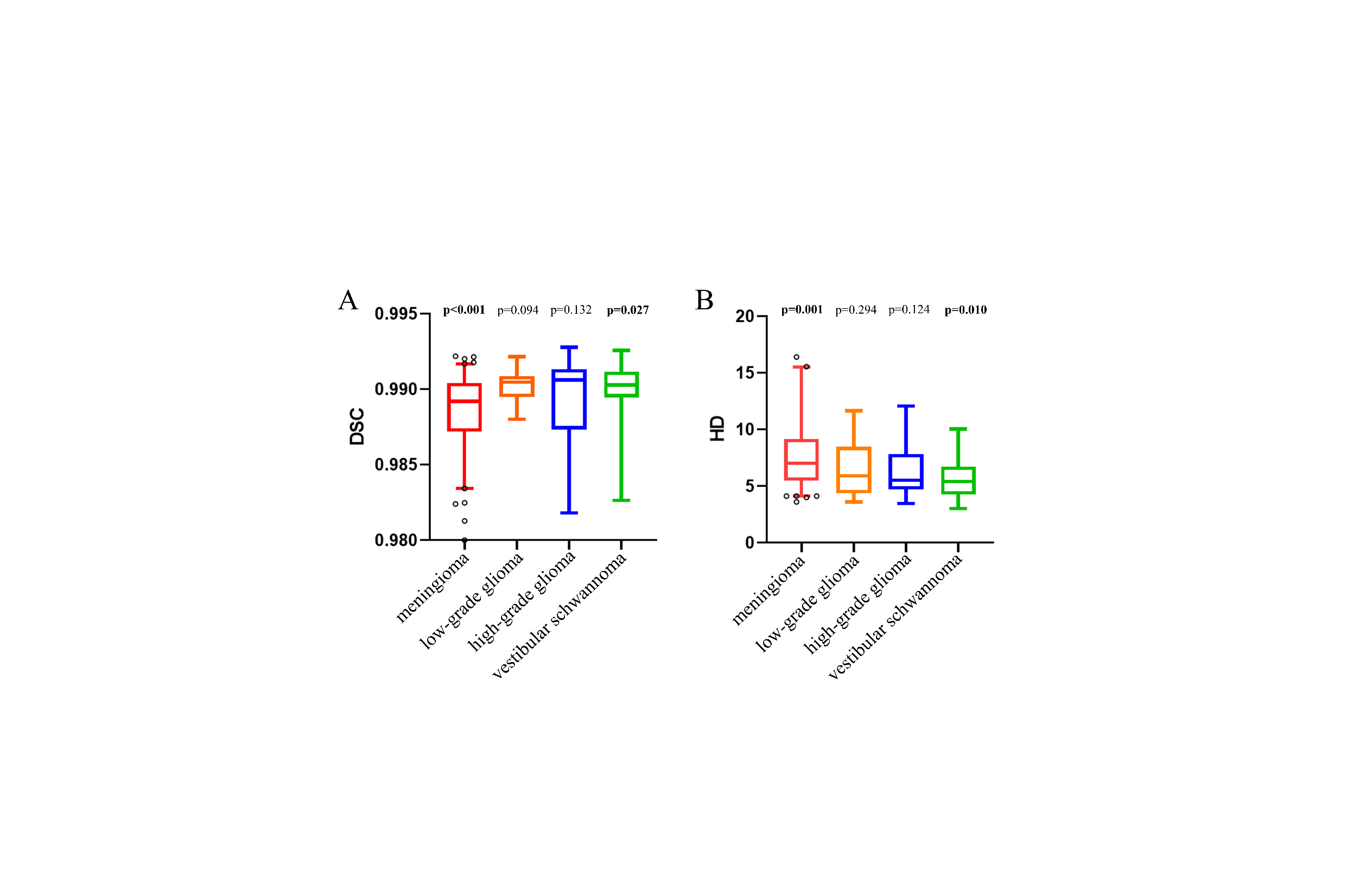
**Supplemental Material 4** Graphs indicting the intra-group analysis of pathological types and tumor characteristics.

**Supplemental Material 4a** Comparison of DSC and HD between the tumor intra-groups for the internal test group using box charts (higher DSC and lower HD indicate better performance). (A) DSCs of meningioma, low-grade glioma, high-grade glioma, and vestibular schwannoma groups. (B) HD of meningioma, low-grade glioma, high-grade glioma, and vestibular schwannoma groups. The p-value is calculated by comparing the DSC and HD for one type of tumor with the rest. DSC: Dice similarity coefficient. HD: Hausdorff Distance.


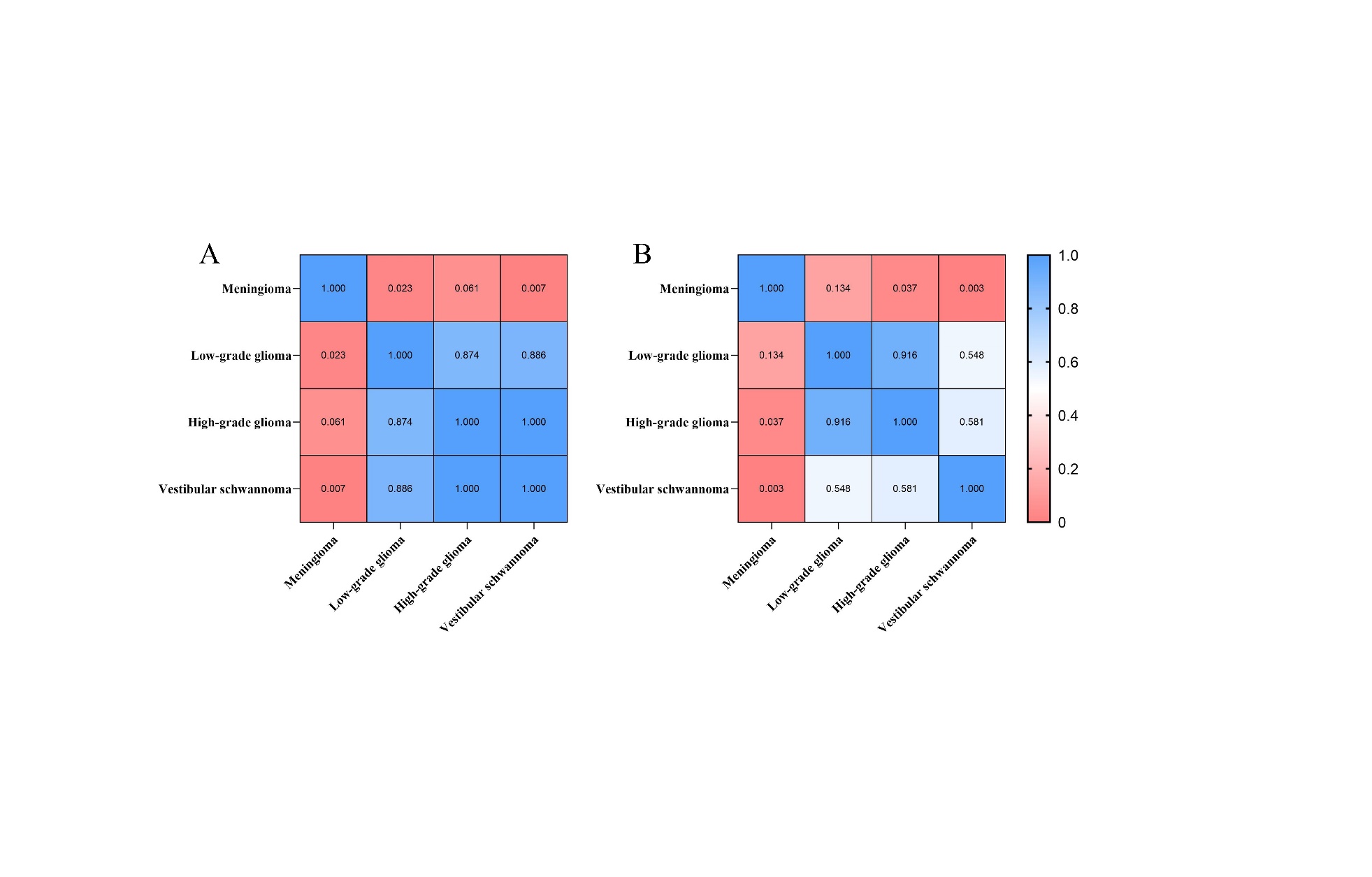
**Supplemental Material 4b** A Heatmap demonstrated the p-values on intra-group analysis of model performance regarding to pathological type. (A) Pairwise p-values of DSC. (B) Pairwise p-values of HD. DSC: Dice similarity coefficient. HD: Hausdorff Distance.


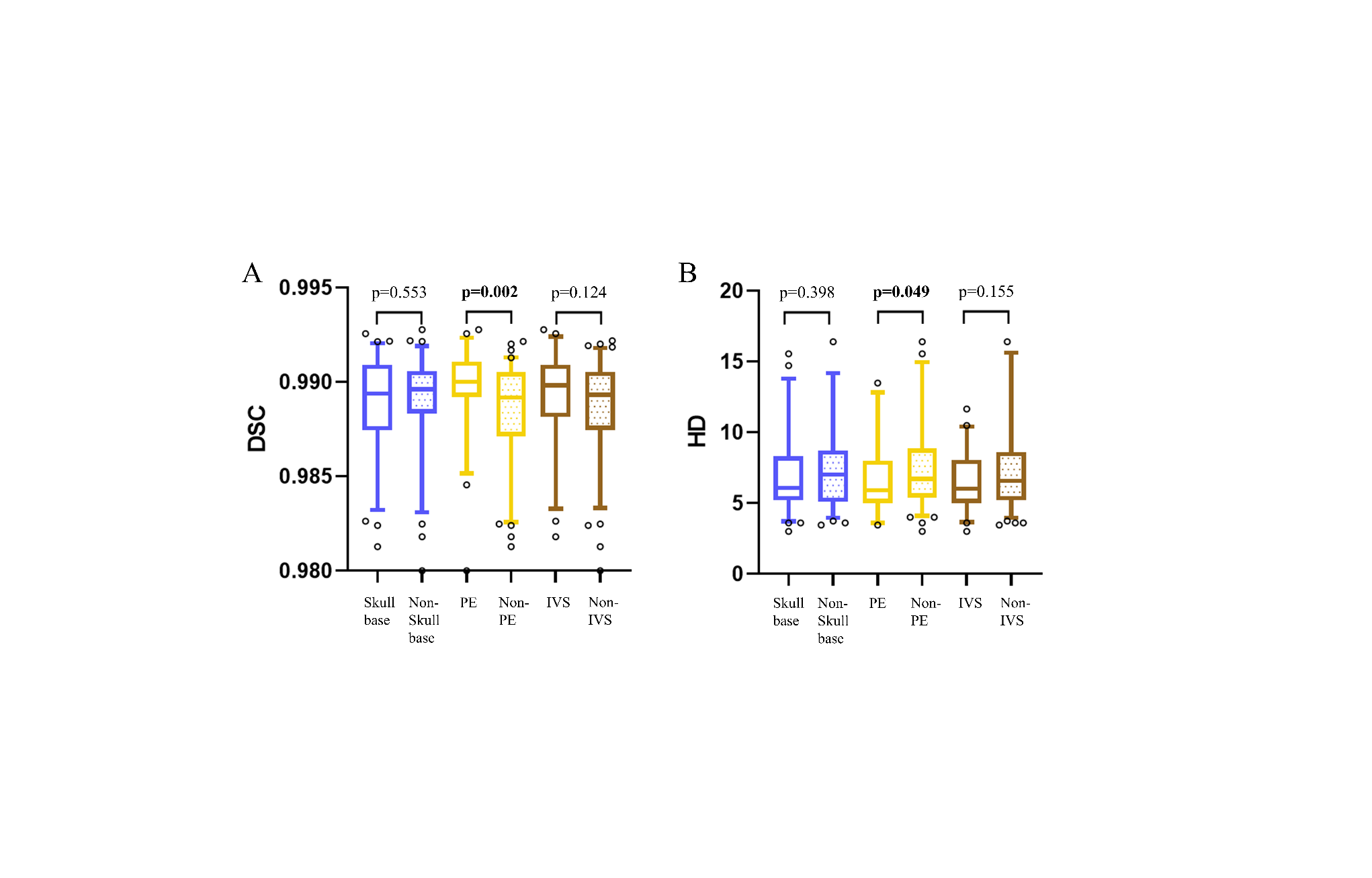
**Supplemental Material 4c** Comparison of DSC and HD between the intra-group for internal test group using box charts (higher Dice indicates better performance and lower HD indicates worse performance). (A) DSCs of Skull base, IVS, and PE groups in the internal test. (B) HD of Skull base, IVS, and PE groups in the internal test. DSC: Dice similarity coefficient. HD: Hausdorff Distance. IVS: invading venous sinus. PE: peritumoral edema.

**Supplemental Material 5** Detailed model performance of nn-Unet, HD-BET, BrainMaGe, and Robex in internal test and external test.

**Supplemental Material 5a** The performance of four brain extraction models on various tumor groups in the internal test.

| Tumor type | Model | DSC | p value | HD (mm) | p value |
| --- | --- | --- | --- | --- | --- |
| Meningioma | nnU-Net | 0.989 (IQR, 0.987-0.990) | <0.001 | 7.000 (IQR, 5.477-9.165) | <0.001 |
|  | HD-BET | 0.984 (IQR, 0.977-0.992) |  | 9.849 (IQR, 7.874-12.280) |  |
|  | BrainMaGe | 0.967 (IQR, 0.951-0.972) |  | 20.760 (IQR, 11.050-39.110) |  |
|  | Robex | 0.957 (IQR, 0.948-0.963) |  | 12.710 (IQR, 11.190-14.480) |  |
| Low-grade glioma | nnU-Net | 0.991 (IQR, 0.990-0.991) | 0.054 | 5.916 (IQR, 4.354-8.475) | <0.001 |
|  | HD-BET | 0.992 (IQR, 0.991-0.996) |  | 9.157 (IQR, 7.228-11.080) |  |
|  | BrainMaGe | 0.951 (IQR, 0.918-0.963) |  | 45.700 (IQR, 27.710-79.410) |  |
|  | Robex | 0.953 (IQR, 0.945-0.959) |  | 13.110 (IQR, 10.620-14.210) |  |
| High-grade glioma | nnU-Net | 0.991 (IQR, 0.987-0.991) | 0.008 | 5.521 (IQR, 4.687-7.849) | <0.001 |
|  | HD-BET | 0.993 (IQR, 0.992-0.996) |  | 8.321 (IQR, 7.348-10.140) |  |
|  | BrainMaGe | 0.955 (IQR, 0.949-0.962) |  | 40.800 (IQR, 30.470-63.190) |  |
|  | Robex | 0.958 (IQR, 0.949-0.964) |  | 11.910 (IQR, 10.44-12.860) |  |
| Vestibular schwannoma | nnU-Net | 0.990 (IQR, 0.990-0.991) | 0.013 | 5.385 (IQR, 4.243-6.708) | <0.001 |
|  | HD-BET | 0.994 (IQR, 0.992-0.995) |  | 7.999 (IQR, 7.053-9.487) |  |
|  | BrainMaGe | 0.947 (IQR, 0.922-0.956) |  | 47.070 (IQR, 21.850-69.890) |  |
|  | Robex | 0.958 (IQR, 0.951-0.966) |  | 10.910 (IQR, 9.750-13.480) |  |

Note.— The p-value is calculated by comparing the performance of nnU-Net with the other three models together. DSC: Dice similarity coefficient. HD: Hausdorff Distance. IQR: interquartile range.

**
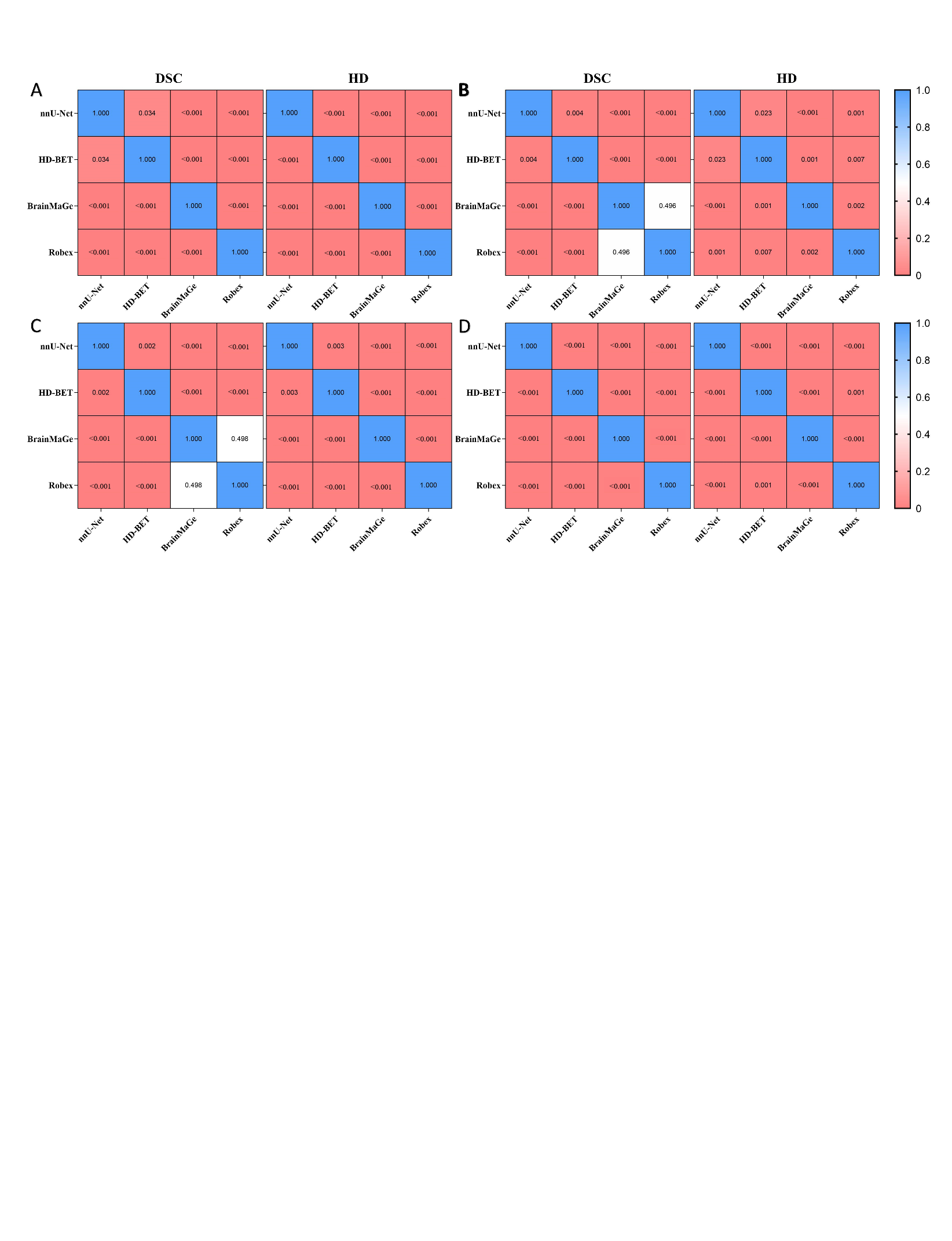
Supplemental Material 5b** A Heatmap indicting the p-values on pairwise analysis of the models in the internal test. (A) meningioma. (B) low-grade glioma. (C) high-grade glioma. (D) vestibular schwannoma. DSC: Dice similarity coefficient. HD: Hausdorff Distance.

**Supplemental Material 5c** The performance of four brain extraction models on various tumor groups in the external test.

| Tomor type | Model | DSC | p value | HD (mm) | p value |
| --- | --- | --- | --- | --- | --- |
| Glioma | nnU-Net | 0.983 (IQR, 0.980-0.986) | <0.001 | 7.000 (IQR, 5.766-8.746) | <0.001 |
|  | HD-BET | 0.978 (IQR, 0.975-0.981) |  | 9.616 (IQR, 7.810-11.080) |  |
|  | BrainMaGe | 0.967 (IQR, 0.961-0.972) |  | 10.150 (IQR, 8.276-14.930) |  |
|  | Robex | 0.953 (IQR, 0.942-0.963) |  | 12.060 (IQR, 9.862-15.000) |  |
| Vestibular schwannoma | nnU-Net | 0.998 (IQR, 0.997-0.999) | <0.001 | 12.86 (IQR, 9.406-15.470) | <0.001 |
|  | HD-BET | 0.984 (IQR, 0.983-0.987) |  | 12.000 (IQR, 10.04-14.090) |  |
|  | BrainMaGe | 0.923 (IQR, 0.913-0.944) |  | 39.240 (IQR, 33.990-46.070) |  |
|  | Robex | 0.961 (IQR, 0.958-0.967) |  | 13.040 (IQR, 12.040-14.370) |  |

Note.— The p-value is calculated by comparing the performance of nnU-Net with the other three models together. DSC: Dice similarity coefficient. HD: Hausdorff Distance. IQR: interquartile range.

**
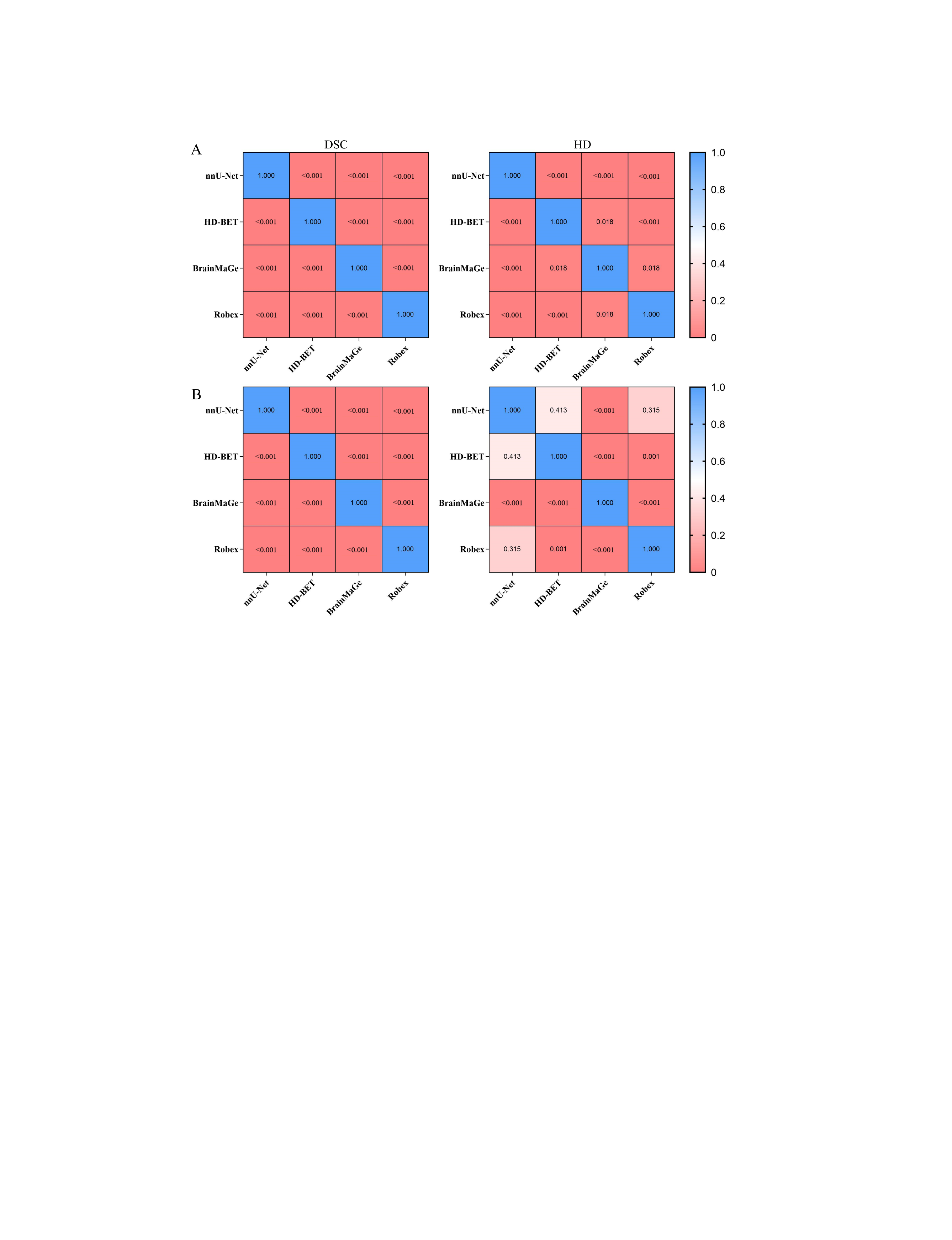
Supplemental Material 5d** A Heatmap indicting the p-values on pairwise analysis of the models in the external test. (A) glioma. (B) vestibular schwannoma. DSC: Dice similarity coefficient. HD: Hausdorff Distance.


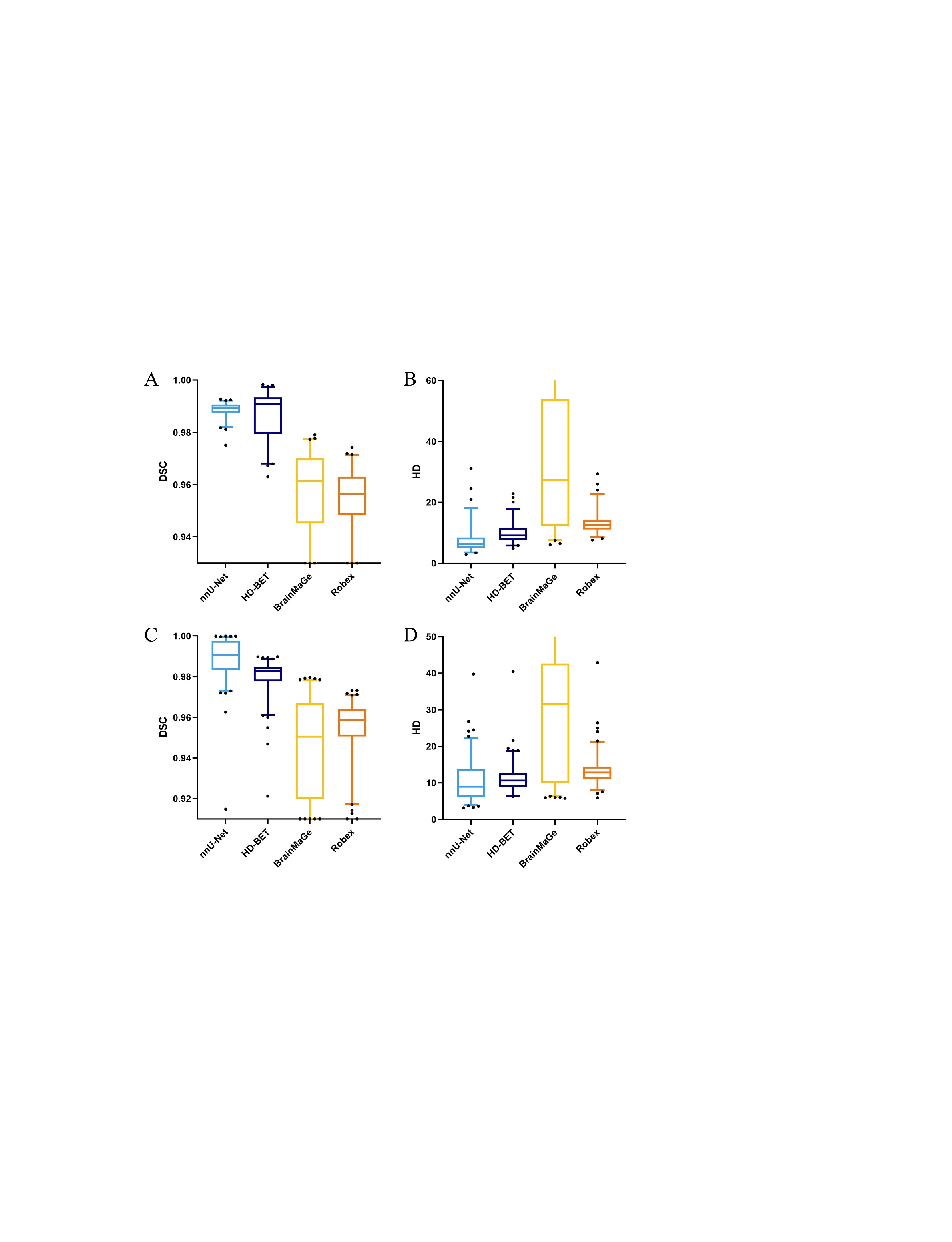


**Supplemental Material 5e** Boxplots illustrating the DSC and HD between the nnU-Net model and three other existing brain extraction models for both the internal and external test datasets. (higher DSC and lower HD indicate better performance). (A-B) DSC and HD of nnU-net, HD-BET, BrainMaGe, and ROBEX groups in internal test. (C-D) DSC and HD of nnU-net, HD-BET, BrainMaGe, and ROBEX groups in external test. DSC: Dice similarity coefficient. HD: Hausdorff Distance. BrainMaGe: Brain Mask Generator. ROBEX: Robust Learning-Based Brain Extraction.


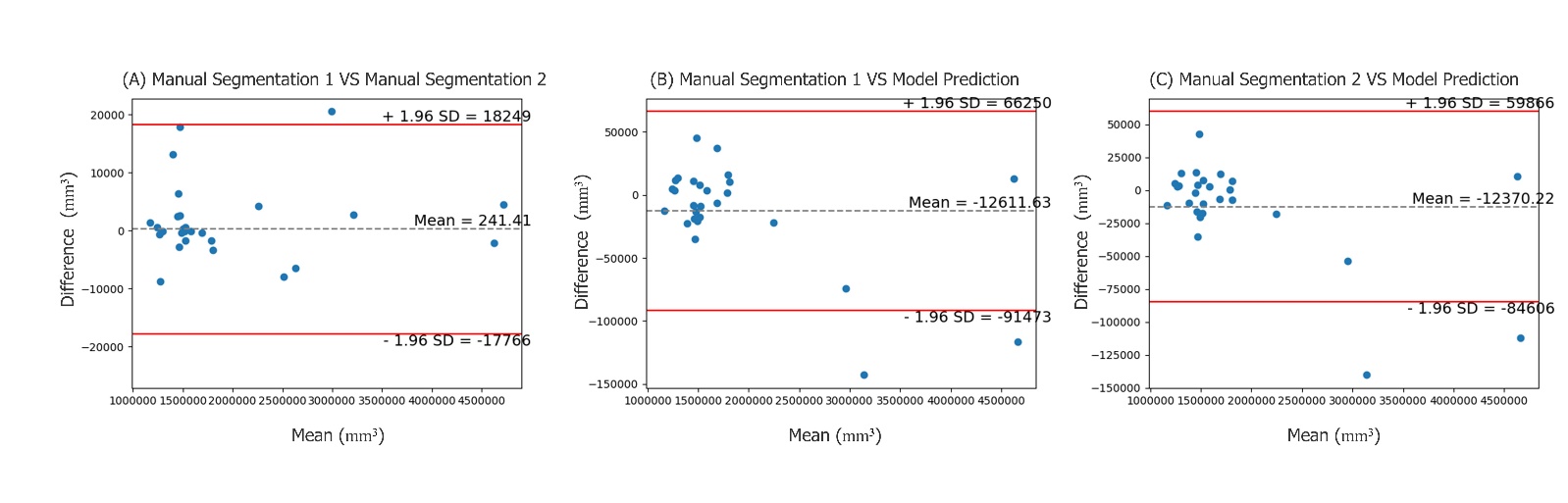
**Supplemental Material 6** One of the Bland-Altman plots of the intraclass repeatability and comparisons among radiologists and model predictions. (A) intra-observer reproducibility of manual segmentation. (B) first time manual segmentation vs model prediction. (C) second time manual segmentation vs model prediction.

**Supplemental Material 7** Summary of the existing brain extraction researches referred to contrast-enhanced T1-weighted MRIs.

| Year | authors | Models | Inputs | Pathological types of tumor data sets | Internal test DSC in T1CE | Internal test HD in T1CE | External test DSC in T1CE | External test HD in T1CE |
| --- | --- | --- | --- | --- | --- | --- | --- | --- |
| 2016 | Jens Kleesiek et al | 3D CNN model | T1WI; T1CE; FLAIR; T2WI | Glioma | 0.95±0.01 | Not Provided | Absent | Absent |
| 2019 | Fabian Isensee et al | HD-BET | T1WI; T1CE; FLAIR; T2WI | Glioma | 0.969 (IQR, 0.961–0.974) | 3.2 mm (IQR, 2.8–4.1 mm) | Absent | Absent |
| 2020 | Siddhesh Thakur et al | 3d-res-u-net | T1WI; T1CE; FLAIR; T2WI | Glioma | UPenn: 0.97±0.00 | UPenn: 2.0±1.18mm | TJU: 0.96±0.01  MDA: 0.95±.01 | TJU: 4.12±2.76mm  MDA: 4.58±2.83mm |
| 2022 | Linmin Pei et al | EnNet | T1WI; T1CE; FLAIR; T2WI | Glioma | 0.9850±0.0171 | 2.6098±2.4814mm | TCIA: 0.9699 ± 0.0016 | TCIA:  4.2099 ± 1.52mm |

Note.—DSC: Dice similarity coefficient. HD: Hausdorff Distance. MRIs: magnetic resonance imagings. T1CE: contrast-enhanced T1-weighted. CNN: Convolutional Neural Network. IQR: interquartile range. TCIA: The Cancer Imaging Archive.
